# The Volume-Outcome Relationship in Retroperitoneal Soft Tissue Sarcoma: Evidence of Improved Short- and Long-Term Outcomes at High-Volume Institutions

**DOI:** 10.1155/2018/3056562

**Published:** 2018-07-24

**Authors:** Sanjay P. Bagaria, Matthew Neville, Richard J. Gray, Emmanuel Gabriel, Jonathan B. Ashman, Steven Attia, Nabil Wasif

**Affiliations:** ^1^Department of Surgery, Section of Surgical Oncology, Mayo Clinic, Jacksonville, FL, USA; ^2^Department of Biostatistics, Mayo Clinic, Scottsdale, AZ, USA; ^3^Department of Surgery, Section of Surgical Oncology, Mayo Clinic, Phoenix, AZ, USA; ^4^Department of Radiation Oncology, Mayo Clinic, Phoenix, AZ, USA; ^5^Division of Hematology/Oncology, Mayo Clinic, Jacksonville, FL, USA; ^6^Robert D. and Patricia E. Kern Center for the Science of Health Care Delivery, Surgical Outcomes Division, Mayo Clinic, Phoenix, AZ, USA

## Abstract

**Background:**

We sought to study the association between RPS case volume and outcomes. Although a relationship has been demonstrated between case volume and patient outcomes in some cancers, such a relationship has not been established for retroperitoneal sarcomas (RPSs).

**Study Design:**

The National Cancer Database (NCDB) was queried for patients undergoing treatment for primary RPS diagnosed between 2004 and 2013. Mean annual patient volume for RPS resection was calculated for all hospitals and divided into low volume (<5 cases/year), medium volume (5–10 cases/year), and high volume (>10 cases/year). Risk-adjusted regression analyses were performed to identify predictors of 30-day surgical mortality, *R*0 margin status, and overall survival (OS).

**Results:**

Our study population consisted of 5,407 patients with a median age of 61 years, of whom 47% were male and 3,803 (70%) underwent surgical resection. Absolute 30-day surgical mortality and *R*0 margin rate following surgery for low-, medium-, and high-volume institutions were 2.4%, 1.3%, and 0.5% (*p*=0.027) and 68%, 65%, and 82%, (*p* < 0.001), respectively. Five-year overall survival rates for low, medium, and high-volume institutions were 56%, 57%, and 66%, respectively (*p* < 0.001). Patients treated at low-volume institutions had a significantly higher risk of 30-day mortality (adjusted OR = 4.66, 95% CI 2.26–9.63) and long-term mortality (adjusted HR = 1.56, 95% CI 1.16–2.11) compared to high-volume institutions.

**Conclusion:**

We demonstrate the existence of a hospital sarcoma service line volume-oncologic outcome relationship for RPS at the national level and provide benchmark data for cancer care delivery systems and policy makers.

## 1. Introduction

Retroperitoneal sarcomas (RPSs) are rare tumors that account for 15–20% of all soft tissue sarcomas. The majority of cases present with localized disease, and therefore, surgical resection is the mainstay of treatment. Extirpations are complex procedures that often require removal of adjacent organs and dissection along critical structures, and as a result, they can be associated with significant morbidity. Furthermore, the efficacy of surgery depends on the ability to achieve negative margins, which requires multidisciplinary preoperative and intraoperative expertise in radiology, pathology, and surgical subspecialties.

Hospital surgical volume is a well-studied measure associated with improved outcomes in multiple tumor subtypes [1–4]. In particular, such a relationship has been reported for complex surgical oncologic procedures such as colectomy, nephrectomy, and pancreatectomy, all of which are commonly performed for RPS [5–7]. As a result, centralization of complex surgical oncologic procedures to high-volume centers has been advocated [1, 2, 4]. The premise is that high-volume centers have the experience and expertise to perform these complex procedures in an optimal manner with correspondingly superior outcomes compared to low-volume centers.

Such a volume-outcome association has not been demonstrated at the national level for RPS, likely due to the rarity of the disease. Nevertheless, due to the multispecialty expertise required to optimally treat these tumors, an association may exist. Our hypothesis is that high-volume centers treating primary RPS have a lower 30-day postoperative mortality, higher margin negative resection rate, and improved overall survival compared to low-volume centers.

## 2. Methods

### 2.1. Data Source

Data from the National Cancer Database (NCDB) were used to conduct this study. The NCDB, a joint program of the Commission on Cancer (CoC) of the American College of Surgeons (ACoS) and the American Cancer Society (ACS), is a nationwide database for more than 1,500 commission-accredited cancer programs in the United States and Puerto Rico. Approximately 70% of all newly diagnosed cases of cancer in the United States are captured at the institutional level and reported to the NCDB. Variables in the database cover demographics, socioeconomic status, tumor stage, treatment received, and hospital characteristics. NCDB data contain no protected health information; hence, this study was exempt from formal IRB review.

### 2.2. Inclusion/Exclusion Criteria

Patients diagnosed with RPS from 2004 to 2013 were identified from the NCDB and constituted our study population (note that patients with extraabdominal sarcoma and gastrointestinal stromal tumors were not included). This time period was chosen to ensure up-to-date coding in the NCDB for the variables of interest for this study and to provide at least 5 years of follow-up for survival analyses.

Two patient cohorts were created. The first comprised all patients diagnosed with RPS irrespective of whether they underwent surgery or not. This group included all stages and represents the institutional experience with RPS. We believe this group better represents any association between volume and long-term survival as it captures multidisciplinary care. The second cohort is a subset of this group comprising only of patients who underwent curative intent surgery. This group was defined to study the association between surgical volume and short-term outcomes. NCDB surgical codes distinguish between curative intent surgery and procedures such as open biopsies. For this surgery-only group, patients with metastatic disease and those not undergoing curative intent surgery were excluded. For both groups, only patients who had all treatment (surgery, radiation therapy, and/or chemotherapy) at the reporting hospital were included in order to provide valid volume-outcome comparisons.

### 2.3. Outcome, Exposure, and Independent Variables

The exposure variable was the hospital volume status. Primary outcome variables were overall survival (OS), surgical margins, and 30-day surgical mortality. Surgical margins were defined as negative (*R*0) or positive (*R*1/*R*2). Independent variables included age, sex, race, insurance status, education, modified Charlson score, primary tumor site, pathologic tumor size, grade, histology, radiation therapy, and chemotherapy. Tumor histology was grouped into clinically significant categories by using ICD codes. Tumor grade was divided into GX (unknown), G1 (well differentiated), G2 (moderately differentiated), and G3 (poorly or undifferentiated).

### 2.4. Hospital Volume Calculations

Average annual volume/hospital of curative intent surgery for RPS was calculated by dividing the total number of surgical resections performed at a hospital by the number of years that data were reported to the NCDB. A histogram of average annual volume/hospital was then plotted, and volume cutoffs were chosen to divide our patient cohorts into three groups: low volume (<5 cases/year), medium volume (5–10 cases/year), and high volume (>10 cases/year) to ensure volume groups with adequate number of patients to enable robust statistical analyses. For analyses involving the all cases cohort, the same volume cutoffs were used to ensure comparability.

### 2.5. Statistical Analyses

Bivariate analyses were initially performed to identify demographic, tumor, and treatment differences between different volume categories using the chi-square test or analysis of variance. Logistic regression analyses were used to model the margin negative resection and 30-day mortality following surgery, and adjusted odds ratios (OR) and 95% confidence intervals (CI) were reported. OS was estimated by the Kaplan–Meier method, and the comparison in the survival curves between different surgical volumes was assessed by the log rank test. Cox regression analyses were used to model overall survival, and adjusted hazard ratios (HR) and 95% CIs were reported. Patients who died within 30 days of surgery were excluded from survival analyses. A *p* value of <0.05 was set as our threshold for statistical significance. The analysis was performed using SAS 9.4 and R 3.3.0.

## 3. Results

### 3.1. Demographics

We identified 5,407 patients with primary RPS who comprised our primary study population (Table 1). The median age was 61 years, and 53% were female. The mean tumor size was 18.5 cm (median = 15.5 cm), and a plurality of tumors were well differentiated (36%). Approximately 76% of patients underwent surgery, 26% received radiation therapy, and 17% received systemic therapy. There were 3,807 patients who underwent surgical resection for curative intent of primary RPS (Table 2) excluding stage-4 cancers. The median age was 62 years, and 53% were female. The median tumor size was 16.8 cm. Most tumors were well differentiated (36%).

Tables 1 and 2 also provide descriptive data stratified by annual volume (<5 cases, 5–10 cases, and >10 cases). The number of high-volume centers in the United States performing >10 primary surgical resections on average annually was only 3/678 (0.4%), while the overwhelming majority were low volume 671/678 (99%). Using the same volume cutoffs for all cases of primary RPS treated increased the high-volume centers to four. Correspondingly, low volume centers treated 83% of all patients and also performed 83% of all curative intent surgery, while the same proportion was 10% and 11% for high-volume centers. Overall, high-volume centers were all academic/research centers, whereas approximately 24% of low-volume centers met that CoC designation. High-volume centers were more likely to provide care to males, Caucasians, those with lower Charlson–Deyo scores, and those who lived in high educational attainment zip codes. The median distance travelled for treatment at a high-volume hospital was 76 miles, compared to 12 miles for a low-volume hospital. High-volume centers were more likely to treat patients whose tumors were larger (17.5 cm versus 15 cm) and of higher grade (58% versus 47%) than low-volume centers. It is important to note that, since the NCDB does not report recurrence, these data only reflect the management of primary RPS and do not provide insight into the management of recurrent RPS.

### 3.2. Short-Term Outcomes (Surgery-Only Cohort)

A total of 82 patients (2.2%) died within 30 days after surgery was performed. The absolute 30-day mortality rates were 0.5%, 1.3%, and 2.4% at high, medium, and low volume centers, respectively. Following adjustment (Table 3), patients undergoing RPS surgery at a low-volume hospital had a greater than fourfold increase in the risk of dying within 30 days of surgery compared to patients undergoing surgery at a high-volume hospital (OR = 4.66, 95% CI 2.26–9.63; *p* < 0.001). On sensitivity analyses, 90-day mortality rates followed a similar trend for absolute and adjusted risk of postoperative mortality.

The overall *R*0 margin rate was 69%. The *R*0 rate was 82%, 65%, and 68% for high-, medium-, and low-volume centers, respectively.  Table 4 displays the multivariable analysis for *R*0 margin rate; low-volume centers were less likely to achieve *R*0 margin status compared to high-volume centers (OR = 0.46, 95% CI 0.31–0.70; *P*=0.0003).

### 3.3. Long-Term Outcomes (All Cases)

**Figure 1:**
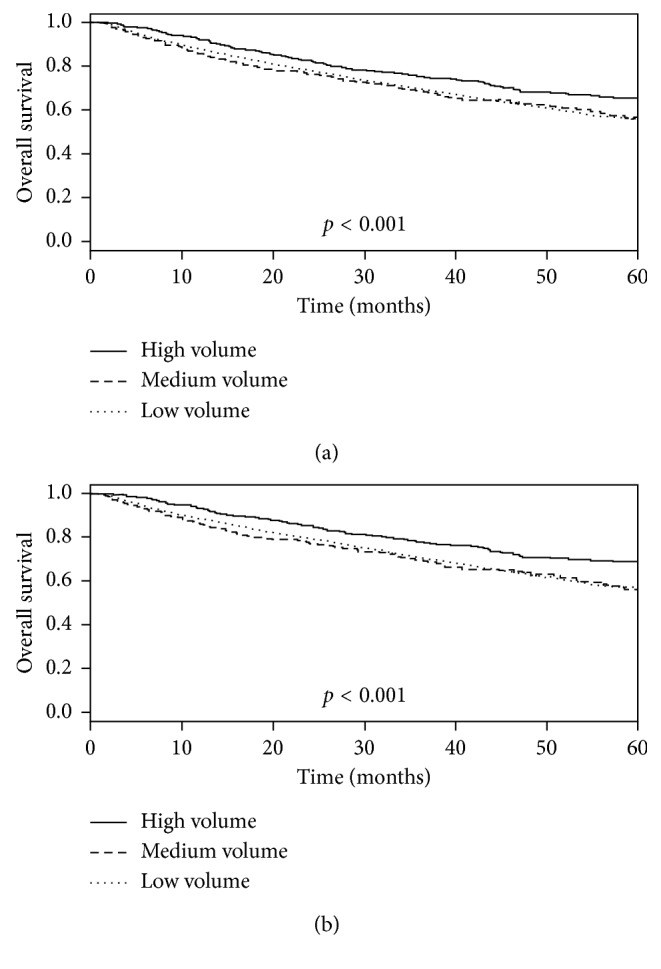
Overall survival stratified by hospital volume. (a) All cases. (b) Surgical cases.

The median follow-up to last contact or death is 37 months. There were 2,282 deaths (42%) from all causes over the duration of the study. For all RPS patients, the 5-year overall survival was 57.1% (95% CI 55.6–58.7%). When stratified by the hospital volume, the 5-year overall survival for high-, medium-, and low-volume centers was 66%, 57%, and 56% (*p* < 0.001; Figure 1(a)). For RPS patients who underwent surgical resection for curative intent, the 5-year overall survival rate was 58.6% (95% CI 56.8–60.5). When stratified by the hospital volume, the 5-year overall survival rates for patients undergoing curative intent surgery in high-, medium-, and low-volume centers was 69%, 56%, and 57%, respectively (*p* < 0.001; Figure 1(b)).

Table 5 displays the multivariable analysis for overall survival. After controlling for patient and tumor variables, patients who were treated at a low-volume hospital had a 52% greater risk of all-cause long-term mortality compared to those treated at a high-volume hospital (HR 1.56, 95% CI 1.16–2.11; *p*=0.0032).

## 4. Discussion

In this study of patients diagnosed with primary RPS, we show that patients treated at high-volume centers were four times less likely to die within 30 days of the procedure, 54% more likely to have an *R*0 margin status, and 52% more likely to be alive by the end of follow-up when compared to patients in low-volume centers. These results are consistent with a strong volume-outcome association for RPS for both short- and long-term outcomes.

Hospital surgical volume has been suggested to be a proxy for superior outcomes for multiple complex surgical oncologic procedures. Although surgical volume in itself impacts outcomes, it is likely that case volume is also associated with the quality of other processes at the institution. Optimal outcomes in this disease require team-based multidisciplinary care including medical oncology, radiation oncology, radiology, and pathology. The rarity of RPS means that tumor boards, compliance to NCCN guidelines, and coordination of survivorship care are all likely to contribute to better oncologic outcomes. For patients who did undergo surgery, the complexity of the surgical procedure requires processes designed to optimize perioperative care and manage complications such as subspecialty trained surgical oncologists, urologists, vascular surgeons, tertiary anesthesiologists, interventional radiologists, and critical care teams to optimize short-term outcomes. It is likely that increased surgical volume allows for a gain in proficiency both within the operating room as well as at the institutional level, which translates into better perioperative outcomes.

Although an argument could be made that our choice of cutoff to define what constitutes a high-volume institution is somewhat arbitrary, and this is consistent with another large study on the subject. The Transatlantic Retroperitoneal Sarcoma Working Group (TARPSWG) has published on 1,007 primary RPS patients treated in eight North American and European sarcoma centers from 2002 to 2011 [8, 9]. These centers are considered high volume and as a group averaged approximately 12 cases/year, similar to the threshold in this study. When we compare high-volume centers identified in the current study to the TARPSWG, we note other similarities in the outcomes measured. The group investigated the safety of resection of primary RPS and reported that Clavien–Dindo ≥3 events occurred in 16% of patients and that the 30-day mortality was 1.8%. In the current study, high-volume centers had a 30-day mortality of 0.5%, suggesting that the cutoff used in the current study may be appropriate to optimize 30-day mortality.

The TARPSWG also studied oncologic outcomes on the same patients and reported an *R*0/*R*1 resection rate of 95%. In the current study, high-volume centers had a comparable *R*0/*R*1 resection rate of 98%; when the negative margin was defined by assessment of microscopic disease; high-volume centers were significantly more likely to achieve *R*0 resection than low-volume centers. However, it should be noted that, since microscopic assessment of the entire tumor surface is not feasible, not all institutions routinely report microscopic margin status. Indeed, approximately 26% of all surgical cases had missing margin data, and therefore, extraction of margin information from pathology reports and subsequent reporting to the NCDB for RPS will need to be improved. Finally, TARPSWG reported a 5-year overall survival rate of 67%, which compares with the 69% seen in our study for high-volume centers. Overall, the comparison with the TARPSWG data suggests that high-volume centers both within and outside of the United States have comparable outcomes.

A review of soft tissue sarcomas identified in the Florida Cancer Data System (FCDS) suggested that high-volume centers—defined as ≥ 5 surgical cases/year—had superior 30-day mortality and 5-year overall survival [10]. Subset analysis of truncal/retroperitoneal sarcomas (*n*=1.745) showed improved 5-year overall survival for high-volume centers (low volume, 32% versus high volume, 36%). Margin status was not available for analysis. When compared to the 5-year overall survival observed at high-volume centers in the current study and in the TARPSWG study (67% and 69%, resp.), the FCDS study reported a lower 5-year overall survival at high-volume centers (36%). This large difference in overall survival highlights the importance of establishing a cutoff (10 versus 5 cases in this instance) that offers better outcomes for RPS patients.

The association between institutional volume and improved outcomes has been used to advocate regionalization of complex procedures to high-volume centers. Even in the absence of health care policy change, this phenomenon had been observed for a variety of cancer types requiring complex surgery [11, 12]. Finks et al. used Medicare data to examine trends in hospital volume and the proportion of patients undergoing surgical resection in high-volume centers. The authors reported that regionalization of complex cancer resections appears to have occurred in the decade following reports that there is an inverse relationship between hospital volume and outcomes [11]. In the context of regionalization, it is important to note that, in our study, only 3 hospitals met the threshold for high volume (>10 surgical cases/year on average) out of a hospital cohort of 678. However, it is also important to note that the data presented in this study refer to treatment of primary RPS and not recurrent disease. Multiple reports from large volume centers suggest that resection of recurrent RPS constitutes approximately 32–44% of their RPS patient population [13–15]. Since the NCDB does not record recurrence and surgery for recurrence, the number of high-volume centers managing and resecting RPS is certainly underestimated by the current data. Nevertheless, the number of high-volume centers appears to be low, and further research will be needed, likely using qualitative or mixed methods, to investigate why significant regionalization has not occurred with regard to RPS. One clue may be the proximity of patients to a high-volume center. The median distance travelled for treatment at a high-volume center was 76 miles compared to 12 miles for a low-volume center. Whether this was due to referral networks, patient education/income insurance contracts, or other factors is unknown.

In this study, only 3 hospitals qualified as high-volume centers. All commission on cancer-approved hospitals are required to report to the NCDB, and these cancer diagnoses account for 70% of new cancers diagnosed in the United States. Since the cancer programs that report to the NCDB include 19 of 20 National Comprehensive Cancer Network (NCCN) hospitals, 33 or 37 NCI-designated cancer centers, and 69 of 121 major inpatient VA hospitals, it is likely that our study captures most of the major sarcoma centers in the US. One likely explanation for only identifying 3 high-volume hospitals is that the current study only reports treatment for primary RPS and not recurrent RPS. Multiple retrospective studies from large sarcoma centers report that treatment of locally recurrent RPS accounts for 24–35% of all RPS cases [13, 14, 16]. It is highly likely that the inclusion of recurrent RPS would increase the number of high-volume centers and that the current analysis underestimates the number of high-volume centers.

A limitation of this study is that it does not provide surgeon-specific volume data. There is evidence that a surgeon's volume is what drives good outcomes, and that when a surgeon relocates, those good outcomes also relocate to the new institution. Whether this is true for rare malignancy such as RPS is not entirely clear. Another limitation is that the NCDB does not contain ECOG performance status, American Society of Anesthesiologists physical status level, and other indicators of functional status. It does contain components of the Charlson Comorbidity Index which allows for a certain degree of adjustment for functional status. The NCDB also does not report the number of organs resected, the number of intestinal anastomoses, and postoperative complications, and therefore, we cannot comment on complexity of the surgery performed at the institutions. Many of the RPS histologic subtypes were unknown. Recent data suggest that RPS histologic subtype influences pattern of failure and death [8]. Whether histologic subtype influenced the results for high-volume centers cannot be determined. The NCDB also does not report on recurrence and disease-specific mortality. RPS has a high rate of local recurrence, and whether institutional volume affects local recurrence and disease-specific death cannot be ascertained. Moreover, the number of patients undergoing treatment for recurrent RPS cannot be determined, and therefore, the actual patient volume per institution is underestimated in this analysis. Finally, since re-resection for recurrent RPS is not captured in this study, and there may be a separate volume-outcome association that may be stronger than the one we observe for primary tumors.

## 5. Conclusions

Despite superior short- and long-term outcomes for high-volume institutions treating RPS, the overwhelming majority of patients continue to be seen at low-volume institutions. While it may be impractical to regionalize all RPS care to the few existing high-volume institutions, there is certainly room for consolidation. Other efforts to disseminate expertise, such as remote tumor boards, centralizing pathology review such as seen in Europe, visits by expert surgeons, and telemedicine may need to be explored.

## Figures and Tables

**Table 1:** Patient characteristics: all patients.

	High volume (*N* = 563)	Medium volume (*N* = 373)	Low volume (*N* = 4471)	Total (*N* = 5407)	*p* value
Age at diagnosis					0.0085
Mean (SD)	59.6 (14.3)	58.5 (14.4)	60.7 (14.2)	60.4 (14.2)	
Median	60.0	59.0	62.0	61.0	
*Q*1, *Q*3	51.0, 70.0	50.0, 68.0	52.0, 71.0	52.0, 71.0	
Range	(19.0–90.0)	(18.0–88.0)	(18.0–90.0)	(18.0–90.0)

Sex					0.0001
Male	304 (54.0%)	193 (51.7%)	2028 (45.4%)	2525 (46.7%)	
Female	259 (46.0%)	180 (48.3%)	2443 (54.6%)	2882 (53.3%)	

Race					0.0010
Black	34 (6.0%)	30 (8.0%)	500 (11.2%)	564 (10.4%)	
Other	29 (5.2%)	26 (7.0%)	271 (6.1%)	326 (6.0%)	
White	500 (88.8%)	317 (85.0%)	3700 (82.8%)	4517 (83.5%)	

Charlson–Deyo score					0.0047
0	472 (83.8%)	307 (82.3%)	3500 (78.3%)	4279 (79.1%)	
1	79 (14.0%)	52 (13.9%)	755 (16.9%)	886 (16.4%)	
2	12 (2.1%)	14 (3.8%)	216 (4.8%)	242 (4.5%)	

Year of diagnosis					0.37
2004	49 (8.7%)	20 (5.4%)	353 (7.9%)	422 (7.8%)	
2005	41 (7.3%)	25 (6.7%)	392 (8.8%)	458 (8.5%)	
2006	56 (9.9%)	37 (9.9%)	404 (9.0%)	497 (9.2%)	
2007	56 (9.9%)	38 (10.2%)	422 (9.4%)	516 (9.5%)	
2008	66 (11.7%)	37 (9.9%)	453 (10.1%)	556 (10.3%)	
2009	64 (11.4%)	34 (9.1%)	452 (10.1%)	550 (10.2%)	
2010	51 (9.1%)	38 (10.2%)	479 (10.7%)	568 (10.5%)	
2011	68 (12.1%)	48 (12.9%)	522 (11.7%)	638 (11.8%)	
2012	59 (10.5%)	59 (15.8%)	508 (11.4%)	626 (11.6%)	
2013	53 (9.4%)	37 (9.9%)	486 (10.9%)	576 (10.7%)	

Facility type					<0.0001
Missing	48	39	364	451	
Community cancer program	0 (0.0%)	1 (0.3%)	230 (5.6%)	231 (4.7%)	
Comprehensive community cancer program	0 (0.0%)	0 (0.0%)	1428 (34.8%)	1428 (28.8%)	
Academic/research program	515 (100.0%)	283 (84.7%)	2023 (49.3%)	2821 (56.9%)	
Integrated network cancer program	0 (0.0%)	50 (15.0%)	426 (10.4%)	476 (9.6%)	

Primary payor					<0.0001
Not insured	7 (1.2%)	10 (2.7%)	181 (4.0%)	198 (3.7%)	
Private insurance	243 (43.2%)	195 (52.3%)	2127 (47.6%)	2565 (47.4%)	
Medicaid	17 (3.0%)	25 (6.7%)	271 (6.1%)	313 (5.8%)	
Medicare	168 (29.8%)	131 (35.1%)	1774 (39.7%)	2073 (38.3%)	
Other government	5 (0.9%)	1 (0.3%)	61 (1.4%)	67 (1.2%)	
Insurance status unknown	123 (21.8%)	11 (2.9%)	57 (1.3%)	191 (3.5%)	

Median income quartiles					0.0078
Missing	36	10	159	205	
<$30,000	36 (6.8%)	41 (11.3%)	547 (12.7%)	624 (12.0%)	
$30,000–$35,999	98 (18.6%)	67 (18.5%)	716 (16.6%)	881 (16.9%)	
$36,000–$45,999	139 (26.4%)	101 (27.8%)	1124 (26.1%)	1364 (26.2%)	
$46,000+	254 (48.2%)	154 (42.4%)	1925 (44.6%)	2333 (44.8%)	

No high school degree (%)					<0.0001
Missing	36	10	159	205	
≥29%	58 (11.0%)	33 (9.1%)	681 (15.8%)	772 (14.8%)	
20–28.9%	96 (18.2%)	91 (25.1%)	961 (22.3%)	1148 (22.1%)	
14–19.9%	125 (23.7%)	96 (26.4%)	994 (23.1%)	1215 (23.4%)	
<14%	248 (47.1%)	143 (39.4%)	1676 (38.9%)	2067 (39.7%)	

Distance to treating center (miles)					<0.0001
Mean (SD)	223.5 (389.7)	68.2 (119.1)	39.5 (136.1)	60.6 (187.7)	
Median	75.9	38.4	12.4	15.4	
*Q*1, *Q*3	22.4, 242.7	15.0, 89.8	5.1, 33.5	5.8, 46.2	
Range	(1.0–4040.1)	(1.0–1495.6)	(1.0–4710.1)	(1.0–4710.1)	

Histologic subtype					<0.0001
Dedifferentiated liposarcoma	160 (28.4%)	81 (21.7%)	755 (16.9%)	996 (18.4%)	
Fibrosarcoma	10 (1.8%)	5 (1.3%)	67 (1.5%)	82 (1.5%)	
Leiomyosarcoma	98 (17.4%)	88 (23.6%)	1069 (23.9%)	1255 (23.2%)	
Liposarcoma	188 (33.4%)	98 (26.3%)	1468 (32.8%)	1754 (32.4%)	
MFH	5 (0.9%)	5 (1.3%)	96 (2.1%)	106 (2.0%)	
MPNST	6 (1.1%)	6 (1.6%)	42 (0.9%)	54 (1.0%)	
Rare/NOS	96 (17.1%)	90 (24.1%)	974 (21.8%)	1160 (21.5%)	

Grade					<0.0001
Missing	97	71	992	1160	
Well differentiated	172 (36.9%)	85 (28.1%)	1256 (36.1%)	1513 (35.6%)	
Mod differentiated	23 (4.9%)	38 (12.6%)	587 (16.9%)	648 (15.3%)	
Poorly differentiated	99 (21.2%)	111 (36.8%)	1019 (29.3%)	1229 (28.9%)	
Undifferentiated	172 (36.9%)	68 (22.5%)	617 (17.7%)	857 (20.2%)	

Tumor size					0.35
Missing	36	17	327	380	
5–10 cm	98 (18.6%)	80 (22.5%)	848 (20.5%)	1026 (20.4%)	
<5 cm	42 (8.0%)	27 (7.6%)	391 (9.4%)	460 (9.2%)	
>10 cm	387 (73.4%)	249 (69.9%)	2905 (70.1%)	3541 (70.4%)	

AJCC stage group					<0.0001
Stage I	180 (32.0%)	90 (24.1%)	1322 (29.6%)	1592 (29.4%)	
Stage II	49 (8.7%)	39 (10.5%)	560 (12.5%)	648 (12.0%)	
Stage III	204 (36.2%)	140 (37.5%)	1278 (28.6%)	1622 (30.0%)	
Stage IV	38 (6.7%)	20 (5.4%)	314 (7.0%)	372 (6.9%)	
AJCC staging not applicable	22 (3.9%)	27 (7.2%)	275 (6.2%)	324 (6.0%)	
AJCC stage group unknown	70 (12.4%)	57 (15.3%)	722 (16.1%)	849 (15.7%)	

Surgery					<0.0001
No	53 (9.4%)	73 (19.6%)	1198 (26.8%)	1324 (24.5%)	
Yes	510 (90.6%)	300 (80.4%)	3273 (73.2%)	4083 (75.5%)	

Radiation					<0.0001
Missing	0	1	79	80	
No	453 (80.5%)	293 (78.8%)	3196 (72.8%)	3942 (74.0%)	
Yes	110 (19.5%)	79 (21.2%)	1196 (27.2%)	1385 (26.0%)	

Chemotherapy					0.0001
Missing	6	11	188	205	
No	469 (84.2%)	270 (74.6%)	3569 (83.3%)	4308 (82.8%)	
Yes	88 (15.8%)	92 (25.4%)	714 (16.7%)	894 (17.2%)	

Last contact or death, months from Dx					0.68
Mean (SD)	41.7 (27.4)	42.3 (31.6)	43.8 (32.2)	43.5 (31.7)	
Median	37.6	35.8	37.2	37.1	
*Q*1, *Q*3	20.0, 58.0	16.8, 61.0	17.9, 64.8	18.1, 63.5	
Range	(0.6–133.1)	(1.2–132.5)	(0.0–142.6)	(0.0–142.6)

Vital status					<0.0001
Dead	177 (31.4%)	151 (40.5%)	1954 (43.7%)	2282 (42.2%)	
Alive	386 (68.6%)	222 (59.5%)	2517 (56.3%)	3125 (57.8%)	

Hospital volume					<0.0001
Mean (SD)	153.7 (46.5)	62.6 (5.0)	14.4 (12.5)	32.2 (47.1)	
Median	134.0	64.0	10.0	13.0	
*Q*1, *Q*3	114.0, 212.0	62.0, 65.0	5.0, 22.0	6.0, 35.0	
Range	(103.0–212.0)	(51.0–68.0)	(1.0–47.0)	(1.0–212.0)	

**Table 2:** Patient characteristics: all surgical cases.

	High volume (*N* = 401)	Medium volume (*N* = 235)	Low volume (*N* = 3167)	Total (*N* = 3803)	*p* value
Age at diagnosis					0.0045
Mean (SD)	59.6 (13.7)	58.7 (14.5)	61.2 (13.9)	60.9 (14.0)	
Median	60.0	60.0	62.0	62.0	
*Q*1, *Q*3	51.0, 69.0	50.0, 69.0	52.0, 71.0	52.0, 71.0	
Range	(20.0–90.0)	(19.0–87.0)	(18.0–90.0)	(18.0–90.0)

Sex					0.0002
Male	216 (53.9%)	130 (55.3%)	1438 (45.4%)	1784 (46.9%)	
Female	185 (46.1%)	105 (44.7%)	1729 (54.6%)	2019 (53.1%)	

Race					0.0010
Black	21 (5.2%)	15 (6.4%)	350 (11.1%)	386 (10.1%)	
Other	22 (5.5%)	18 (7.7%)	184 (5.8%)	224 (5.9%)	
White	358 (89.3%)	202 (86.0%)	2633 (83.1%)	3193 (84.0%)	

Charlson–Deyo score					0.0061
0	336 (83.8%)	195 (83.0%)	2444 (77.2%)	2975 (78.2%)	
1	55 (13.7%)	34 (14.5%)	565 (17.8%)	654 (17.2%)	
2	10 (2.5%)	6 (2.6%)	158 (5.0%)	174 (4.6%)	

Year of diagnosis					0.20
2004	30 (7.5%)	12 (5.1%)	253 (8.0%)	295 (7.8%)	
2005	31 (7.7%)	19 (8.1%)	259 (8.2%)	309 (8.1%)	
2006	40 (10.0%)	18 (7.7%)	271 (8.6%)	329 (8.7%)	
2007	43 (10.7%)	18 (7.7%)	310 (9.8%)	371 (9.8%)	
2008	51 (12.7%)	22 (9.4%)	306 (9.7%)	379 (10.0%)	
2009	44 (11.0%)	22 (9.4%)	314 (9.9%)	380 (10.0%)	
2010	36 (9.0%)	22 (9.4%)	350 (11.1%)	408 (10.7%)	
2011	38 (9.5%)	42 (17.9%)	389 (12.3%)	469 (12.3%)	
2012	44 (11.0%)	36 (15.3%)	359 (11.3%)	439 (11.5%)	
2013	44 (11.0%)	24 (10.2%)	356 (11.2%)	424 (11.1%)	

Facility type					<0.0001
Missing	31	23	230	284	
Community cancer program	0 (0.0%)	0 (0.0%)	121 (4.1%)	121 (3.4%)	
Comprehensive community cancer program	0 (0.0%)	0 (0.0%)	913 (31.1%)	913 (25.9%)	
Academic/research program	370 (100.0%)	212 (100.0%)	1579 (53.8%)	2161 (61.4%)	
Integrated network cancer program	0 (0.0%)	0 (0.0%)	324 (11.0%)	324 (9.2%)	

Primary payor					<0.0001
Not insured	2 (0.5%)	9 (3.8%)	116 (3.7%)	127 (3.3%)	
Private insurance	175 (43.6%)	109 (46.4%)	1482 (46.8%)	1766 (46.4%)	
Medicaid	6 (1.5%)	18 (7.7%)	189 (6.0%)	213 (5.6%)	
Medicare	102 (25.4%)	96 (40.9%)	1300 (41.0%)	1498 (39.4%)	
Other government	3 (0.7%)	1 (0.4%)	43 (1.4%)	47 (1.2%)	
Insurance status unknown	113 (28.2%)	2 (0.9%)	37 (1.2%)	152 (4.0%)	

Median income quartiles					0.0002
Missing	24	11	119	154	
<$30,000	25 (6.6%)	14 (6.3%)	387 (12.7%)	426 (11.7%)	
$30,000–$35,999	61 (16.2%)	49 (21.9%)	521 (17.1%)	631 (17.3%)	
$36,000–$45,999	93 (24.7%)	61 (27.2%)	807 (26.5%)	961 (26.3%)	
$46,000+	198 (52.5%)	100 (44.6%)	1333 (43.7%)	1631 (44.7%)	

No high school degree quartiles (%)					0.0001
Missing	24	11	119	154	
≥29%	43 (11.4%)	20 (8.9%)	473 (15.5%)	536 (14.7%)	
20–28.9%	62 (16.4%)	55 (24.6%)	694 (22.8%)	811 (22.2%)	
14–19.9%	85 (22.5%)	61 (27.2%)	701 (23.0%)	847 (23.2%)	
<14%	187 (49.6%)	88 (39.3%)	1180 (38.7%)	1455 (39.9%)	

Distance to treating center (miles)					<0.0001
Mean (SD)	259.0 (433.2)	85.3 (149.5)	42.5 (145.9)	68.0 (208.0)	
Median	80.4	49.5	13.7	17.2	
*Q*1, *Q*3	22.9, 306.5	22.3, 100.3	5.4, 38.3	6.2, 50.8	
Range	(1.0–4040.1)	(1.0–1495.6)	(1.0–4710.1)	(1.0–4710.1)	

Histologic subtype					<0.0001
Dedifferentiated liposarcoma	125 (31.2%)	69 (29.4%)	585 (18.5%)	779 (20.5%)	
Fibrosarcoma	6 (1.5%)	4 (1.7%)	45 (1.4%)	55 (1.4%)	
Leiomyosarcoma	64 (16.0%)	38 (16.2%)	715 (22.6%)	817 (21.5%)	
Liposarcoma	143 (35.7%)	64 (27.2%)	1078 (34.0%)	1285 (33.8%)	
MFH	2 (0.5%)	4 (1.7%)	70 (2.2%)	76 (2.0%)	
MPNST	3 (0.7%)	2 (0.9%)	25 (0.8%)	30 (0.8%)	
Rare/NOS	58 (14.5%)	54 (23.0%)	649 (20.5%)	761 (20.0%)	

Grade					<0.0001
Missing	62	53	617	732	
Well differentiated	133 (39.2%)	53 (29.1%)	919 (36.0%)	1105 (36.0%)	
Mod differentiated	15 (4.4%)	19 (10.4%)	426 (16.7%)	460 (15.0%)	
Poorly differentiated	57 (16.8%)	53 (29.1%)	774 (30.4%)	884 (28.8%)	
Undifferentiated	134 (39.5%)	57 (31.3%)	431 (16.9%)	622 (20.3%)	

Tumor size					0.11
Missing	29	6	206	241	
5–10 cm	56 (15.1%)	55 (24.0%)	556 (18.8%)	667 (18.7%)	
<5 cm	29 (7.8%)	17 (7.4%)	236 (8.0%)	282 (7.9%)	
>10 cm	287 (77.2%)	157 (68.6%)	2169 (73.3%)	2613 (73.4%)	

AJCC stage group					0.016
Stage I	128 (31.9%)	63 (26.8%)	989 (31.2%)	1180 (31.0%)	
Stage II	38 (9.5%)	29 (12.3%)	409 (12.9%)	476 (12.5%)	
Stage III	158 (39.4%)	96 (40.9%)	1044 (33.0%)	1298 (34.1%)	
AJCC staging not applicable	17 (4.2%)	18 (7.7%)	196 (6.2%)	231 (6.1%)	
AJCC stage group unknown	60 (15.0%)	29 (12.3%)	529 (16.7%)	618 (16.3%)	

Margins					0.0001
Missing	153	43	810	1006	
Grossly positive (*R*2)	6 (2.4%)	6 (3.1%)	128 (5.4%)	140 (5.0%)	
Microscopically positive (*R*1)	40 (16.1%)	61 (31.8%)	621 (26.3%)	722 (25.8%)	
Negative (*R*0)	202 (81.5%)	125 (65.1%)	1608 (68.2%)	1935 (69.2%)	

30-day mortality					0.027
Patient alive or died more than 30 days after surgery performed	399 (99.5%)	232 (98.7%)	3090 (97.6%)	3721 (97.8%)	
Patient died 30 or fewer days after surgery performed	2 (0.5%)	3 (1.3%)	77 (2.4%)	82 (2.2%)	

90-day mortality					0.0012
Patient alive or died more than 90 days after surgery performed	396 (98.8%)	220 (93.6%)	3000 (94.7%)	3616 (95.1%)	
Patient died 90 or fewer days after surgery performed	5 (1.2%)	15 (6.4%)	167 (5.3%)	187 (4.9%)	

Hospital volume					<0.0001
Mean (SD)	143.5 (38.4)	60.9 (12.1)	12.8 (11.2)	29.5 (44.0)	
Median	108.0	53.0	8.0	11.0	
*Q*1, *Q*3	108.0, 185.0	53.0, 78.0	4.0, 18.0	5.0, 32.0	
Range	(108.0–185.0)	(51.0–78.0)	(1.0–45.0)	(1.0–185.0)	

**Table 3:** Logistic regression analysis of 30-day postoperative mortality.

Variable	95% Wald			
	Odds ratio	Confidence limits		*p* value
Age	1.06	1.04	1.09	<0.0001

Female (reference = male)	0.92	0.61	1.40	0.71

Race (reference = white)				
Black	1.16	0.56	2.39	0.69
Other	0.74	0.23	2.37	0.61

Charlson–Deyo (reference = 2)				
0	1.13	0.65	1.95	0.66
1	1.67	0.79	3.50	0.18

Hospital volume (reference = high)				
Medium	2.73	0.65	11.51	0.17
Low	4.66	2.26	9.63	<0.0001

**Table 4:** Logistic regression analysis of *R*0 margin status.

Variable	95% Wald			
	Odds ratio	Confidence limits		*p* value
Age	0.98	0.98	0.99	<0.0001

Female (reference = male)	1.11	0.94	1.31	0.215

Race (reference = white)				
Black	1.20	0.90	1.60	0.21
Other	0.98	0.69	1.40	0.93

Charlson–Deyo (reference = 2)				
0	0.86	0.69	1.07	0.19
1	0.76	0.51	1.13	0.17

Hospital volume (reference = high)				
Medium	0.38	0.21	0.70	0.0019
Low	0.46	0.31	0.70	0.0003

**Table 5:** Cox regression model for overall survival: all patients.

Variable	95% Wald			
	Odds ratio	Confidence limits		*p* value
Age	1.02	1.02	1.03	<0.0001

Female (reference = male)	0.79	0.72	0.87	<0.0001

Race (reference = white)				
Black	0.81	0.69	0.96	0.015
Other	0.88	0.71	1.09	0.23

Insurance (reference = private)				
Medicaid	1.38	1.11	1.70	0.0034
Medicare	1.14	1.00	1.30	0.056
No insured	1.38	1.07	1.79	0.014
Other government	1.18	0.75	1.86	0.47
Unknown	1.23	0.85	1.78	0.28

No high school degree (reference ≥ 29%)				
<14%	0.77	0.67	0.90	0.0007
14–19.9%	0.86	0.73	1.00	0.055
20–28.9%	0.92	0.79	1.08	0.30

Charlson–Deyo (reference = 2)				
0	0.72	0.58	0.88	0.0011
1	0.81	0.65	1.01	0.055

Tumor size (reference = <5 cm)				
5–10 cm	1.02	0.83	1.25	0.85
>10 cm	1.43	1.19	1.73	0.0001

Grade (reference = grade 3)				
Grade 1	0.34	0.28	0.40	<0.0001
Grade 2	0.52	0.44	0.61	<0.0001
Unknown	0.64	0.56	0.73	<0.0001

Margins (reference = *R*0)				
*R*1	1.31	1.15	1.49	<0.0001
*R*2	2.18	1.73	2.75	<0.0001
Unknown	1.61	1.44	1.80	<0.0001

Surgery (reference = yes)				
No	0.93	0.83	1.04	0.21

Radiation (reference = yes)				
No	0.80	0.72	0.90	<0.0001

Chemotherapy (reference = yes)				
No	1.26	1.11	1.42	0.0004

Hospital volume (reference = high)				
Medium	1.44	0.98	2.10	0.064
Low	1.57	1.16	2.11	0.0032

## Data Availability

The data used to support the findings of this study are available from the corresponding author upon request.
